# Nutritional supplementation alters associations between one-carbon metabolites and cardiometabolic risk profiles in older adults: a secondary analysis of the Vienna Active Ageing Study

**DOI:** 10.1007/s00394-021-02607-y

**Published:** 2021-07-08

**Authors:** Nicola A. Gillies, Bernhard Franzke, Barbara Wessner, Barbara Schober-Halper, Marlene Hofmann, Stefan Oesen, Anela Tosevska, Eva-Maria Strasser, Nicole C. Roy, Amber M. Milan, David Cameron-Smith, Karl-Heinz Wagner

**Affiliations:** 1 Liggins Institute, University of Auckland, Auckland, New Zealand; 2 Riddet Institute, Palmerston North, New Zealand; 3 Research Platform Active Ageing, University of Vienna, Althanstrasse 14, 1090 Vienna, Austria; 4 Department of Sports Medicine, Exercise Physiology and Prevention, University of Vienna, Vienna, Austria; 5 Internal Medicine III, Division of Rheumatology, Medical University of Vienna, Vienna, Austria; 6 Institute for Physical Medicine and Rehabilitation, Kaiser Franz Josef Hospital – Social Medical Center South, Vienna, Austria; 7 Food, Nutrition and Health, AgResearch, Hamilton, New Zealand; 8 The High-Value Nutrition National Science Challenge, Auckland, New Zealand; 9 Department of Human Nutrition, University of Otago, Dunedin, New Zealand; 10 Singapore Institute for Clinical Sciences, Agency for Science, Technology, and Research, Singapore, Singapore; 11 Department of Nutritional Sciences, University of Vienna, Vienna, Austria

**Keywords:** Choline, Homocysteine, Nutritional supplement, Older adults, One-carbon metabolism

## Abstract

**Purpose:**

Cardiovascular diseases and cognitive decline, predominant in ageing populations, share common features of dysregulated one-carbon (1C) and cardiometabolic homeostasis. However, few studies have addressed the impact of multifaceted lifestyle interventions in older adults that combine both nutritional supplementation and resistance training on the co-regulation of 1C metabolites and cardiometabolic markers.

**Methods:**

95 institutionalised older adults (83 ± 6 years, 88.4% female) were randomised to receive resistance training with or without nutritional supplementation (Fortifit), or cognitive training (control for socialisation) for 6 months. Fasting plasma 1C metabolite concentrations, analysed by liquid chromatography coupled with mass spectrometry, and cardiometabolic parameters were measured at baseline and the 3- and 6-month follow-ups.

**Results:**

Regardless of the intervention group, choline was elevated after 3 months, while cysteine and methionine remained elevated after 6 months (mixed model time effects, *p* < 0.05). Elevated dimethylglycine and lower betaine concentrations were correlated with an unfavourable cardiometabolic profile at baseline (spearman correlations, *p* < 0.05). However, increasing choline and dimethylglycine concentrations were associated with improvements in lipid metabolism in those receiving supplementation (regression model interaction, *p* < 0.05).

**Conclusion:**

Choline metabolites, including choline, betaine and dimethylglycine, were central to the co-regulation of 1C metabolism and cardiometabolic health in older adults. Metabolites that indicate upregulated betaine-dependent homocysteine remethylation were elevated in those with the greatest cardiometabolic risk at baseline, but associated with improvements in lipid parameters following resistance training with nutritional supplementation. The relevance of how 1C metabolite status might be optimised to protect against cardiometabolic dysregulation requires further attention.

**Supplementary Information:**

The online version contains supplementary material available at 10.1007/s00394-021-02607-y.

## Introduction

Metabolic dysregulation, leading to cardiovascular diseases, diabetes and cognitive decline are major causes of morbidity and mortality in the ageing population [1, 2]. One central metabolic pathway impaired with ageing is that of one-carbon (1C) metabolism [3, 4]. Traditionally identified on the basis of elevated homocysteine (Hcy), 1C metabolism is central to aspects of both cognitive [5, 6] and cardiometabolic health [7, 8].

**Fig. 1:**
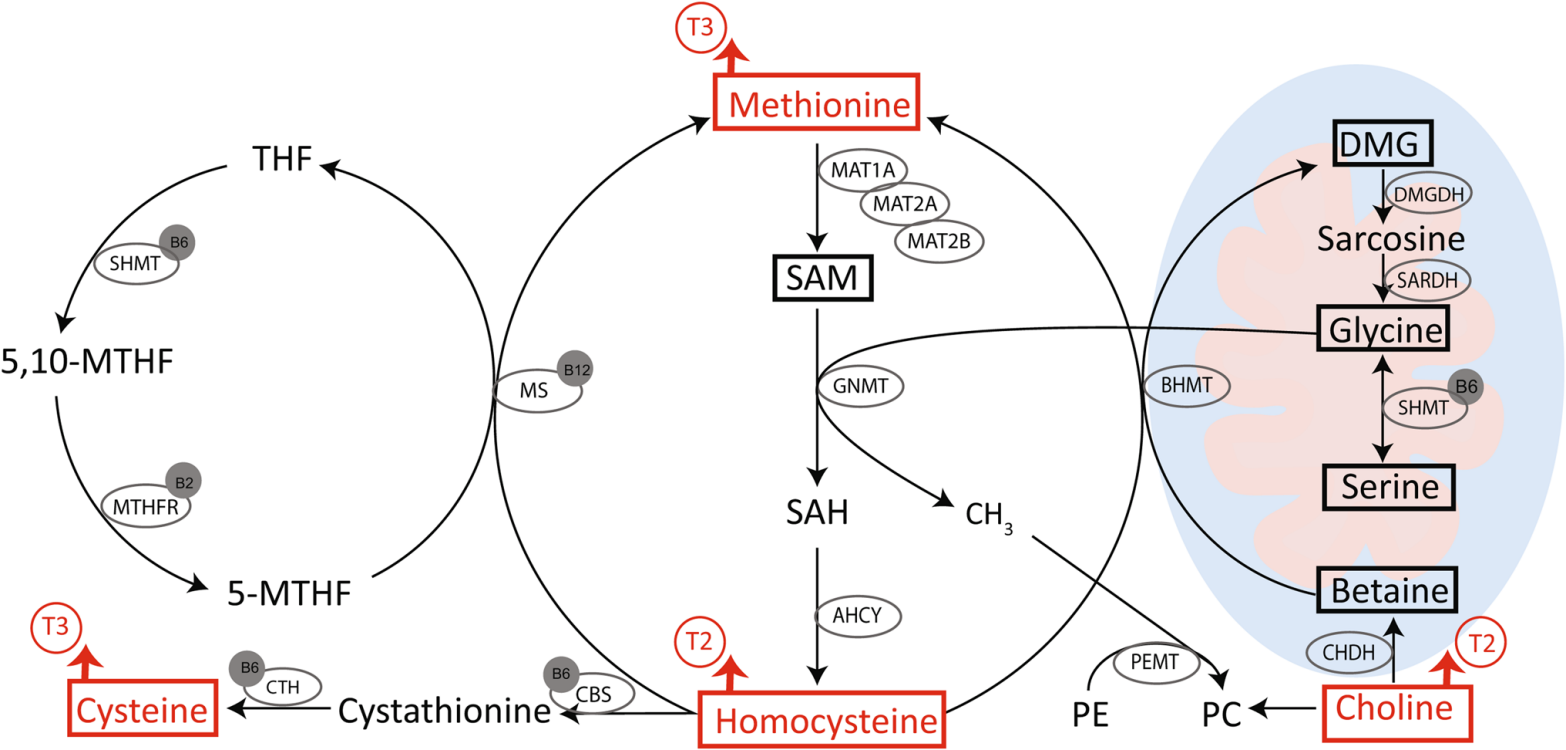
Overview of one-carbon metabolism, and the impact of intervention with resistance training (RT), resistance training with supplementation (RTS), or cognitive training (CT) for 6 months on one-carbon metabolites. Metabolites analysed in this paper are highlighted in bold and outlined in a box, B vitamins acting as co-enzymes are in a grey circle, and enzymes are outlined in an oval. A red arrow indicates a change from baseline to follow-up (↑, increase; ↓, decrease) and time point (T2, 3-month; T3, 6-month) for metabolites where a significant time effect (*p* < 0.05) was found in linear mixed models. No changes according to the intervention group were found for any metabolites (interaction, *p* > 0.05). *AHCY* *S*-adenosylhomocysteine hydrolase, *BHMT* betaine-homocysteine methyltransferase, *CBS* cystathionine *β*-synthase, *CHDH* choline dehydrogenase, *CTH* cystathionine λ-lyase, *DMG* dimethylglycine, *GNMT* glycine *N-*methyltransferase, *MAT* methionine adenosyltransferase, *MS* methionine synthase, *PC* phosphatidylcholine, *PE* phosphatidylethanolamine, *PEMT* phosphatidylethanolamine methyltransferase, *SAH* *S*-adenosylhomocysteine, *SAM* *S*-adenosylmethionine, *SARDH* sarcosine dehydrogenase, *SHMT* serine hydroxymethyltransferase (SHMT1, cytosolic; SHMT2, mitochondrial), *THF* tetrahydrofolate

Hcy is at a critical branch point in 1C metabolism, with circulating concentrations dependent upon competing actions of the 1C cycle. Hcy is synthesised from the conversion of methionine to *S*-adenosylmethionine (SAM) and *S*-adenosylhomocysteine (SAH), and is remethylated through either folate and vitamin B_12_ or betaine-dependent pathways to support the methionine cycle, or is removed through the vitamin B_6_-dependent transsulfuration pathway to form cysteine [9] (Fig. 1). There is a shift towards analysing a more comprehensive 1C metabolite profile to provide greater insights into the coordination of circulating Hcy.

There is substantive evidence that supports the association of 1C metabolites beyond Hcy with cardiometabolic health. Cysteine is proposed as an obesogenic amino acid [10] and elevated glycine conversely with a favourable cardiometabolic profile [11]. Of particular interest is choline and its downstream metabolites, betaine and dimethylglycine (DMG). These metabolites reflect Hcy remethylation by betaine-homocysteine methyltransferase (BHMT). Choline plays a critical role in lipid metabolism, as phosphatidylcholine is required for very low-density lipoprotein synthesis and hepatic lipid transport. Evidence supports divergent associations between choline and betaine and markers of cardiometabolic health. Elevated choline concentrations are typically associated with an unfavourable metabolic profile, and conversely, elevated betaine with a more protective profile for indices of anthropometry, lipid metabolism, and insulin sensitivity [12–15].

Those in their oldest years of life represent a vulnerable group, with an increased risk of dysregulated 1C metabolism [4] and cardiometabolic disease markers [2], yet the relationship between these processes is not well understood. Although the pathways of 1C metabolism are tightly interconnected, there is little evidence from studies that consider metabolites beyond distinct, targeted 1C metabolites (e.g. Hcy or choline and betaine alone). Furthermore, nutritional supplementation becomes more frequent with advancing age to support dietary adequacy and counteract age-related functional decline [16, 17]. Resistance training (RT) is another important lifestyle intervention particularly in elderly subjects, shown to improve cardiometabolic parameters [18] and 1C metabolism with evidence of circulating Hcy reduction [19, 20], and favourably impact related health outcomes such as cognition [21, 22]. Given the overlap of 1C metabolism and cardiometabolic markers in multiple related health outcomes, there is potential to provide various health benefits to older adults. However, the impact of RT and nutritional supplementation containing B vitamins on profiles of 1C metabolites and cardiometabolic parameters requires further investigation in an elderly population.

Thus, in this secondary analysis of the Vienna Active Ageing Study, we undertook a targeted approach to better understand the relationship between 1C metabolism and cardiometabolic health in the elderly. First, by exploring 6-month shifts in 1C metabolites and cardiometabolic markers, and their correlation, in the context of lifestyle interventions including RT with or without nutritional supplementation (containing protein and micronutrients, including folic acid, vitamins B_6_ and B_12_). Second, by investigating the cross-sectional association between markers of cardiometabolic health and 1C metabolites and the influence of B vitamin status.

## Methodology

### Study design

The present article reports on data from the Vienna Active Ageing Study [23], which uses a randomised, controlled, observer-blind design. Participants were randomly assigned to three parallel intervention groups—RT, RT with nutritional supplementation (RTS), or cognitive training (CT), which acted as the control group. Blood samples and anthropometric measurements were taken at baseline, and after 3 and 6 months of intervention. Informed written consent was obtained from all participants before they were enrolled in the trial following the Declaration of Helsinki. The Vienna Active Ageing Study was approved by the ethics committee of the City of Vienna (EK-11-151-0811) and registered at ClinicalTrials.gov, NCT01775111. This article reports on secondary exploratory outcomes; the primary outcome of the Vienna Active Ageing Study was to evaluate the effect of RT or RTS on functional parameters, including markers of chromosomal damage [24], oxidative stress [25], and DNA damage [26]. The secondary outcomes reported in this analysis include the assessment of plasma 1C metabolite concentrations and markers of cardiometabolic health.

### Subjects

Over six months, 117 institutionalised older men and women (aged 65–98 years) were recruited to participate in the Vienna Active Ageing Study from five senior residences in the area of Vienna (Curatorship of the Viennese Retirement Homes). At the six-month follow-up, 95 participants were included for analysis in the current study, and a detailed participant flow diagram describing the loss of participants during follow-up has previously been reported [23]. Participants were eligible to participate if they were mentally (Mini Mental State Examination ≥ 23) [27] and physically (Short Physical Performance Battery > 4) stable. Inclusion and exclusion criteria have previously been described in detail [23]. Briefly, participants were sedentary (< 1 h of physical activity or exercise per week), aged ≥ 65 years, and referring to the recommendations of the American Heart Association [28], were free of diseases that pose contraindication to medical training therapy, including serious cardiovascular diseases, diabetic retinopathy, and regular use of cortisone-containing drugs. Subjects were excluded if they performed regular resistance training (> once/week in the last six months before inclusion) or were using dietary supplements and were not willing to abstain during the intervention.

### Study intervention

#### Resistance training

Groups receiving the training-based intervention (RT and RTS) performed two sessions of RT per week. The training protocol was developed in line with the guidelines of the American College of Sports Medicine for RT with older adults [29]. Exercise sessions were conducted on non-consecutive days and supervised by a sport scientist who recorded attendance. The training program has been described in detail by Oesen et al. [23]. Briefly, each session lasted approximately one hour, consisting of an initial ten-minute warm-up, a 30–40 min strength training using exercise bands and a chair, and a ten-minute cooldown, and the intensity and volume of the exercise were progressively increased.

#### Resistance training with nutritional supplementation

Participants in the RTS group followed the same training protocol as those in the RT group and additionally consumed a nutritional supplement every morning as well as directly after the bi-weekly training session. The nutritional supplement was a single 150 ml serve of FortiFit (Nutricia, Vienna, Austria), a supplement which is freely available to purchase and designed to prevent sarcopenia in older adults. Of note, each 150 kcal serve of this supplement provided 200 µg of folic acid, 3.0 µg of vitamin B_12_, 750 µg of vitamin B_6_, 55 mg of choline, and 20.7 g of protein. Complete details of the composition of this nutritional supplement can be found in Supplementary Table 1. Participants were instructed to consume the supplement at breakfast each morning.

#### Cognitive training

The CT group served as the control group to minimise the bias of being part of a social group activity, as those in the RT and RTS groups received twice a week during training sessions. Those in the CT group performed coordinative or cognitive tasks [30], which were mainly memory training and finger dexterity exercises in a sitting position.

Participants in all intervention groups were instructed to maintain regular food intake, which was checked with 24 h diet recalls when blood samples were taken at three- and six-month follow-ups.

### Cardiometabolic parameters

Waist and hip circumference, body mass and body height were measured following standardised anthropometric procedures. Body mass was measured to the nearest 0.1 kg (BWB 700, Tanita, Amsterdam, Netherlands) and height was measured to the nearest 0.5 cm using a portable stadiometer (SECA Model 217, Seca GmbH & Co. KG, Hamburg, Germany). Body mass index (BMI) was calculated as body mass relative to height in meters squared (kg/m^2^).

Lipid profile (low-density lipoprotein cholesterol (LDL-C), high-density lipoprotein cholesterol (HDL-C), total/HDL-C) and triglycerides), high-sensitive insulin, plasma glucose (FPG) and glomerular filtration rate (GFR) were analysed immediately after blood sampling at a routine laboratory (study lab GmbH, Vienna, Austria). Renal function is a determinant of circulating Hcy concentrations [31, 32], and GFR was therefore used as a confounding variable in regression models. To determine Homeostatic Model Assessment of Insulin Resistance (HOMA-IR) as a marker of insulin resistance, fasting plasma glucose (mg/dl) and insulin (IU/ml) values were converted into mmol/L (glucose) and pmol/L (insulin), and then entered into the validated HOMA2 calculator (HOMA2 v2.2.3 © β, Diabetes Trials Unit, University of Oxford).

### One-carbon metabolites

Following an overnight fast, blood samples were collected into EDTA-coated vacutainers. Samples were placed on ice prior to separation of plasma for analysis [33], and then separated into aliquots and stored in Eppendorf tubes at  − 80 °C until required for analysis. Samples were thawed only immediately before analysis. Ultra-high performance liquid chromatography coupled with mass spectrometry was performed to determine plasma concentrations of 11 metabolites (betaine, choline, DMG, *S*-adenosylhomocysteine (SAH), and SAM) and amino acids (cysteine, cystathionine, glycine, Hcy, methionine, and serine) involved in the regulation of 1C metabolism. Product/precursor ratios were calculated to provide insight into pathway regulation for betaine/choline, and DMG/betaine, which have previously been used as an index of endogenous betaine synthesis and its use, allowing inference of betaine-dependent remethylation of Hcy [34, 35].

Samples were randomised to ensure a balance of intervention group and sex across batches. The methods have been reported in detail elsewhere[36]. Briefly, plasma samples were prepared using an automated robotic liquid handling system (Eppendorf epMotion® 5075vt, Hamburg, Germany). First, 300 μl of 1% formic acid in methanol was pipetted into a 96-well IMPACT® protein precipitation plate (Phenomenex, Torrance, California, USA). Next, all standards (100 μl), quality controls (100 μl) and standards (100 μl) were spiked with 20 μl of internal standard solution, agitated for 5 min (800 rpm), then filtered into a (2 mL) 96-well square collection plate (Phenomenex, Torrance, California, USA) by applying a vacuum (450 mbar). 100 μl of Tris (2-carboxyethyl) phosphine was then dispensed into each well to allow for the separate quantification of Hcy and cysteine.

Three sets of quality control samples were included to assess recovery of standards and reproducibility of samples. Metabolites were considered acceptable if standard recoveries were between 80 and120%, and coefficients of variance were below 20%. Cystathionine and SAH did not satisfy these requirements and were excluded from further analysis, as were SAM samples from one of three batches (34%, *n* = 76). For samples where a peak was not detected, a missing value was calculated as half of the minimum value of each batch [37]. A missing value was not calculated for the SAM samples that were excluded from the analysis.

### B vitamin status

Plasma vitamin B_12_ and erythrocyte folate status were analysed using a radioimmunoassay technique, according to Müllner et al. [38]*.* Standard curves were drawn and sample values calculated according to the protocol provided by the kit producer (MP Biomedicals, Germany). Coefficient of variance was calculated as 5.5% for folate and 5.6% for vitamin B_12_. Inadequate plasma vitamin B_12_ and erythrocyte folate status was defined as < 150 pmol/L and < 340 nmol/L, respectively, according to recommendations by the World Health Organization [39, 40].

### Dietary intake

Dietary intake was assessed by interview-based 24 h recalls, which were performed at baseline and after 6 months. The evaluation of the records was performed using the nutritional software NUT.S (Dato Denkwerkzeuge, Vienna, Austria), which is based on the German Food Composition Database Version II.3 (Berlin, Germany) but was adapted for Austrian eating habits through the addition of typical Austrian recipes.

### Statistical analysis

Before analysis, normality was assessed graphically. Due to a skewed distribution, HOMA-IR, DMG and the DMG/betaine ratio were log-transformed for analysis. Non-transformed values were used to construct tables and graphs of summary statistics. All statistical analyses were performed using R 3.6.3 statistical software [41]. Unless otherwise specified, Alpha was set at *p* ≤ 0.05, and data presented as mean (standard deviation).

Baseline differences in markers of metabolic health and B vitamin status between males and females were assessed by Students *t *test, and 1C metabolites by linear mixed models to correct for batch effect as a random factor. Baseline correlations between markers of metabolic health and 1C metabolites were assessed by Spearman rank correlation. Changes in markers of metabolic health and 1C metabolites were analysed by linear mixed models, where subject and batch effect (1C metabolites only) were fit as a random factor, and time and intervention were fit as fixed factors, including an interaction term between time and intervention. For significant interaction terms, the Sidak adjustment was applied to correct for multiple comparisons. To evaluate associations between shifts in 1C metabolites and markers of metabolic health, and the effect of the intervention received on this, multiple regression modelling was performed. Change (baseline—follow-up) in each metabolic marker was set as the dependent variable, and change in each metabolite was set as the independent variable in the following models. Model 1—unadjusted; Model 2—adjusted (age, sex, GFR, baseline metabolite status, and BMI for non-anthropometric-dependent variables); Model 3—adjusted model including an interaction term between the independent variable (change in metabolite concentration) and the intervention group.

## Results

### Participant characteristics

This secondary analysis of the Vienna Active Ageing Study included 95 participants at baseline, of which 67 had data available for 1C metabolites and cardiometabolic parameters at the six-month follow-up. Participants had an average age of 83 (± 6) years and were predominantly female (88.4%), which reflects the gender distribution of this age group near their statistical life expectancy in the houses of the Curatorship of Viennese Retirement homes (Table 1).

**Table 1:** Baseline status of one-carbon metabolites, cardiometabolic parameters and B vitamins according to sex

Characteristic	Variable	Total (*n* = 95)	Males (*n* = 11)	Females (*n* = 84)	*p *value
	Age (years)	83.1 (5.8)	83.4 (5.2)	82.4 (6.7)	0.110
Metabolic Health	Body mass index (kg/m^2^)	29.1 (4.8)	26.9 (3.4)	29.4 (4.9)	0.045^*^
	Waist/Hip ratio	0.86 (0.07)	0.93 (0.04)	0.85 (0.07)	< 0.001^*^
	Serum LDL Cholesterol (mmol/L)	3.17 (0.97)	2.77 (0.98)	3.22 (0.96)	0.178
	Serum HDL cholesterol (mmol/L)	1.65 (0.44)	1.51 (0.36)	1.67 (0.44)	0.200
	Serum Total/HDL-cholesterol ratio	3.47 (1.01)	3.23 (0.66)	3.50 (1.04)	0.240
	Serum Triglycerides (mmol/L)	1.31 (0.51)	1.09 (0.34)	1.33 (0.52)	0.051
	Plasma glucose (mmol/L)	5.71 (1.10)	5.43 (0.84)	5.74 (1.13)	0.280
	HOMA-IR	2.41 (2.30)	1.65 (1.18)	2.51 (2.39)	0.064
One-carbon metabolites	Betaine (µM)	30.8 (9.36)	35.0 (10.5)	30.3 (9.13)	0.193
	Choline (µM)	14.3 (2.59)	14.4 (3.49)	14.3 (2.48)	0.477
	Cysteine (µM)	103 (21.9)	104 (13.6)	102 (22.8)	0.539
	DMG (µM)	2.84 (1.26)	2.74 (1.25)	2.85 (1.27)	0.749
	Glycine (µM)	195 (48.1)	157 (25.6)	200 (48.2)	0.003^*^
	Homocysteine (µM)	2.20 (1.07)	2.00 (1.12)	2.23 (1.07)	0.943
	Methionine (µM)	22.45 (3.19)	24.3 (3.21)	22.2 (3.12)	0.061
	*S-*adenosylmethionine (nM)	56.6 (16.8)	53.3 (22.0)	57.1 (16.1)	0.555
	Serine (µM)	95.8 (26.6)	79.9 (16.1)	97.9 (27.0)	0.050
	Betaine/choline	2.20 (0.69)	2.44 (0.46)	2.16 (0.71)	0.211
	DMG/betaine	0.10 (0.05)	0.09 (0.06)	0.10 (0.05)	0.940
B vitamin status	Plasma Vitamin B_12_ (pmol/L)	380 (333)	460 (410)	370 (324)	0.456
	Prevalence of vitamin B_12_ inadequacy^a^	21%	20%	30%	0.479
	Erythrocyte folate (nmol/L)	181 (107)	185 (95.4)	181 (109)	0.962
	Prevalence of folate inadequacy^b^	93%	94%	89%	0.589
Dietary intake	Energy (kcal)	1556 (354)	1705 (298)	1544 (357)	0.176
	Protein (% energy intake)	16.2	17.3	16.1	
	Fat (% energy intake)	35.0	34.4	35.1	
	Carbohydrate (% energy intake)	48.8	48.3	48.8	
	Fibre (g)	21.7 (30.6)	21.7 (7.0)	21.7 (31.8)	0.176
	Protein (g/kg/body weight)	0.83 (0.30)	0.85 (0.24)	0.83 (0.27)	0.701
	Folate (µg)	261 (124)	300 (121)	257 (125)	0.450
	Riboflavin (mg)	1.17 (0.41)	1.02 (0.35)	1.18 (0.42)	0.347
	Vitamin B_6_ (mg)	1.56 (0.78)	1.73 (0.47)	1.55 (0.80)	0.205
	Vitamin B_12_ (µg)	5.05 (3.03)	5.59 (3.64)	5.01 (2.99)	0.618

Data presented as mean (standard deviation). All biochemical measures are fasting samples. Abbreviations: *DMG* dimethylglycine

^a^Inadequate plasma vitamin B_12_ was defined as < 150 pmol/L according to the World Health Organization[40]

^b^Inadequate erythrocyte folate was defined as < 340 nmol/L according to the World Health Organization[39]

*Indicates a significant difference (*p* < 0.05) between sex for variables at baseline

### Differences in B vitamins, one-carbon metabolites and cardiometabolic parameters according to sex.

At baseline, females had a higher BMI (*p* = 0.045), but lower waist/hip ratio (*p* ≤ 0.001) than their male counterparts, with a trend towards higher serum triglycerides (*p* = 0.051) and HOMA-IR, or greater insulin resistance, (*p* = 0.064). Minor differences were seen between 1C metabolite status at baseline; females had higher plasma glycine concentrations at baseline (*p* = 0.003), and a trend towards higher plasma serine (*p* = 0.050) and lower methionine (*p* = 0.061) concentrations. No difference in B vitamin status (plasma vitamin B_12_ and erythrocyte folate) or adequacy was found between males and females. Notably, there was a high prevalence of folate inadequacy in the total population (93% with concentrations < 340 nmol/L, the adequacy threshold recommended by the World Health Organization [39]) (Table 1).

### Baseline correlations between one-carbon metabolites and cardiometabolic parameters

According to Spearman correlation analyses, cysteine concentration was positively correlated with BMI (*ρ* = 0.202) and the waist/hip ratio (*ρ* = 0.223). The waist/hip ratio was also positively correlated with both methionine (*ρ* = 0.219) and glycine (*ρ* = 0.398). HDL-C was positively correlated with glycine (*ρ* = 0.345), and the total/HDL-C ratio with DMG (*ρ* = 0.208) and DMG/betaine (*ρ* = 0.230). Choline metabolites were also correlated with triglycerides, with an inverse correlation between both betaine (*ρ* =  − 0.300) and betaine/choline (*ρ* = − 0.287), while a positive correlation was found with both DMG (*ρ* = 0.233) and DMG/betaine (*ρ* = 0.335). An inverse correlation with betaine (*ρ* = − 0.254) and positive correlation with DMG/betaine (*ρ* = 0.207) was also found for HOMA-IR, but FPG was not correlated with 1C metabolites (Table 2).

**Table 2:** Baseline correlation analysis between one-carbon metabolites and cardiometabolic parameters

	BMI	Waist/hip ratio	LDL-Cholesterol	HDL-Cholesterol	Total/HDL-Cholesterol	Triglycerides	Glucose	HOMA-IR
Betaine	− 0.191*	0.180*	0.076	0.196*	− 0.116	− 0.300**	− 0.086	− 0.254**
Choline	− 0.135	0.050	0.031	0.032	− 0.035	− 0.053	− 0.201*	− 0.104
Cysteine	0.202**	0.223**	− 0.094	− 0.167	0.061	0.158	0.053	0.091
DMG	0.043	− 0.022	0.122	− 0.083	0.208**	0.233**	− 0.182	0.077
Glycine	0.071	− 0.398	0.044	0.345***	− 0.173*	− 0.128	− 0.125	− 0.063
Homocysteine	0.170	0.065	− 0.080	− 0.145	0.045	0.097	0.101	0.080
Methionine	− 0.046	0.219**	− 0.099	− 0.004	− 0.127	− 0.199*	0.039	− 0.088
*S-*adenosylmethionine	0.143	0.134	− 0.032	− 0.238	0.206	− 0.150	0.083	0.142
Serine	0.109	− 0.088	0.078	0.074	0.004	− 0.049	− 0.092	− 0.046
Betaine/choline	− 0.100	0.146	− 0.019	0.180*	− 0.147	− 0.287**	0.043	− 0.176
DMG/Betaine	0.144	− 0.057	0.081	− 0.160	0.230**	0.335***	− 0.068	0.207**

Correlation coefficients presented according to Spearman correlation analysis. *p < 0.100, **p < 0.05, ***p < 0.01. Abbreviations: *DMG* dimethylglycine, *SAM* *S*-adenosylmethoinine

### Changes in one-carbon metabolites and cardiometabolic parameters according to intervention group

Across all intervention groups, an increase of the waist/hip ratio (time effect, *p* = 0.008) and LDL-C (time effect, *p* = 0.028) was found at the six-month follow-up, while a decrease in HOMA-IR was found for the three-month follow-up, but was not sustained at the 6-month follow-up (time effect, *p* ≤ 0.001). In the control group, we also found a reduction in FPG at the three-month follow-up, but again was not sustained at the 6-month follow-up (interaction, *p* = 0.006). Plasma choline concentrations increased at the 3-month follow-up only (time effect, *p* = 0.013), while Hcy, methionine and cysteine concentrations remained elevated at the six-month follow-up (time effect, *p* ≤ 0.001). An interaction effect was found for serine (*p* = 0.024). However, post hoc comparisons were not significant upon applying the Sidak correction (Table 3, Fig. 1). Changes in B vitamin concentrations from baseline to follow-up have been presented elsewhere [24]. Of relevance to the present findings, an increase in erythrocyte folate and plasma vitamin B_12_ concentrations was reported to increase in the RTS group only [24].

**Table 3:** Baseline, three- and six-month status of one-carbon metabolites and cardiometabolic parameters according to intervention group

Parameter	Marker	RT			RTS			CT			*p *value	
		Baseline	FU-3mo	FU-6mo	Baseline	FU-3mo	FU-6mo	Baseline	FU-3mo	FU-6mo	Time	Interaction
Metabolic Health	BMI	29.0 (3.62)	28.9 (3.65)	29.0 (3.59)	29.3 (6.02)	27.7 (3.36)	29.1 (4.91)	28.9 (4.68)	27.4 (4.85)	28.3 (5.17)	0.154	0.982
	WHR	0.85 (0.06)	0.86 (0.06)	0.88 (0.06)^b^	0.85 (0.07)	0.84 (0.06)	0.86 (0.08)^b^	0.87 (0.07)	0.85 (0.07)	0.88 (0.05)^b^	0.008^*^	0.201
	LDL-C	123 (43.4)	121 (45.3)	128 (38.7)^b^	124 (33.5)	121 (36.8)	131 (41.9)^b^	124 (36.5)	120 (37.3)	126 (39.8)^b^	0.028^*^	0.974
	HDL-C	64.2 (16.5)	65.9 (16.9)	62.3 (13.9)	66.1 (15.6)	64.7 (18.2)	64.7 (17.2)	63.0 (19.0)	61.3 (14.5)	62.3 (16.8)	0.977	0.939
	Total/HDL-C	3.47 (1.18)	3.30 (0.82)	3.57 (0.88)	3.40 (0.91)	3.45 (0.95)	3.63 (1.12)	3.54 (0.92)	3.45 (0.81)	3.53 (0.83)	0.304	0.744
	Triglycerides	114 (49.0)	111 (47.6)	120 (46.0)	117 (37.8)	127 (48.3)	128 (42.7)	117 (48.6)	114 (50.3)	115 (41.5)	0.790	0.657
	Glucose	106 (24.1)	101 (20.2)	103 (17.8)	96.0 (10.5)	102 (15.2)	94.1 (12.3)	106 (20.7)	94.9 (14.6)^b^	97.8 (11.8)	0.001^*^	0.006^a^
	HOMA-IR	2.66 (2.99)	1.05 (1.56)^b^	2.60 (3.36)	2.24 (1.42)	1.06 (0.98)^b^	2.38 (1.93)	2.32 (2.20)	0.85 (0.92)^b^	2.06 (1.77)	< 0.001^*^	0.989
One-carbon metabolites	Betaine	29.3 (8.16)	32.8 (11.1)	30.9 (10.3)	31.1 (10.0)	32.2 (6.20)	32.9 (8.26)	32.2 (9.92)	33.0 (7.46)	30.0 (8.08)	0.623	0.316
	Choline	13.9 (2.66)	15.4 (2.50)^b^	13.9 (3.47)	14.3 (2.29)	15.4 (2.89)^b^	15.4 (3.08)	14.6 (2.81)	16.5 (3.01)^b^	15.5 (3.37)	0.013^*^	0.312
	Cysteine	102 (21.2)	109 (19.0)	115 (20.7)^b^	102 (23.3)	105 (18.2)	112 (23.0)^b^	103 (21.9)	111.5 (25.3)	114 (22.8)^b^	< 0.001^*^	0.932
	DMG	2.53 (0.82)	2.50 (0.73)	2.42 (0.83)	3.14 (1.68)	2.67 (1.24)	3.34 (1.79)	2.88 (1.15)	3.20 (2.25)	2.80 (1.20)	0.624	0.231
	Glycine	200 (53.6)	204 (69.1)	207 (70.4)	203 (51.8)	215 (62.3)	200 (42.2)	184 (36.4)	183 (30.6)	185 (34.7)	0.323	0.812
	Hcy	2.23 (0.91)	2.39 (0.99)	2.52 (0.87)^b^	2.18 (0.89)	2.25 (0.91)	2.09 (0.68)^b^	2.19 (1.38)	2.58 (1.21)	2.64 (1.14)^b^	0.009^*^	0.264
	Methionine	22.7 (3.09)	24.6 (3.67)^b^	24.1 (3.95)^b^	22.6 (3.36)	25.7 (4.95)^b^	25.0 (3.99)^b^	23.1 (3.21)	23.1 (3.39)^b^	22.8 (3.47)^b^	< 0.001^*^	0.274
	Serine	98.0 (26.7)	97.2 (19.6)	97.7 (26.5)	100 (30.9)	83.4 (13.7)	91.8 (25.1)	89.9 (21.3)	85.6 (13.3)	89.9 (16.6)	0.501	0.024^a^
	Bet/Cho	2.12 (0.44)	2.13 (0.54)	2.26 (0.61)	2.20 (0.73)	2.15 (0.57)	2.16 (0.53)	2.27 (0.84)	2.04 (0.49)	1.95 (0.39)	0.395	0.255
	DMG/Bet	0.10 (0.05)	0.08 (0.04)	0.09 (0.04)	0.10 (0.05)	0.09 (0.05)	0.11 (0.08)	0.10 (0.06)	0.10 (0.07)	0.10 (0.04)	0.609	0.434

Values presented as mean (standard deviation). *p* values are presented for the main effect of time and interaction effect of time x intervention received. Folate and vitamin B_12_ concentrations have been reported elsewhere [24]

Abbreviations: *Bet/Cho* Betaine/choline, *BMI* body mass index, *CT* cognitive training (control), *DMG* dimethylglycine, *DMG/Bet* DMG/betaine, *FU-3mo* 3-months follow-up, *FU-6mo* 6-months follow-up, *Hcy* homocysteine, *HDL-C* HDL-cholesterol, *LDL-C* LDL-cholesterol, *RT* resistance training, *RTS* resistance training with supplementation, *WHR* waist/hip ratio

*Indicates a significant main effect of time according to analysis with mixed models (*p* < 0.05)

^a^Indicates a significant interaction effect according to analysis with mixed models (*p* < 0.05)

^b^Indicates which time points differ from baseline according to post hoc comparisons adjusted with the Sidak correction (*p* < 0.05)

### Longitudinal associations between changes in one-carbon metabolites and cardiometabolic parameters

Increasing Hcy concentrations were associated with a rise in the waist/hip ratio in both unadjusted (*β* = 0.020, *p* = 0.008) and adjusted models (*β* = 0.020, *p* = 0.042), while a positive association between shifts in cysteine concentrations and the waist/hip ratio was significant in an unadjusted model only (*β* = 8.2^–4^, *p* = 0.035). Increasing DMG concentrations were associated with a decline of BMI (*β* =  − 0.410, *p* = 0.009) and LDL-C (*β* = − 10.8, *p* = 0.031), although only that with BMI remained significant when adjusted for confounding variables (*β* = − 0.400, *p* = 0.038). Increasing DMG was also associated with a rise in HOMA-IR in an adjusted model only (*β* = 0.72, *p* = 0.018). A positive association was also found between shifts in the ratio of total/HDL-C and DMG/betaine, a marker of upregulated betaine-dependent Hcy remethylation, in unadjusted (*β* = 6.17, *p* = 0.034) and adjusted (*β* = 8.74, *p* = 0.014) models (Table 4, Supplementary Table 2).

**Table 4:** Summary of the relationship between shifts in one-carbon metabolites and cardiometabolic parameters from baseline to 6-months follow-up according to linear regression analysis

Metabolite	Model	BMI		Waist/Hip ratio		LDL-C		HDL-C		Total/HDL-C		Triglycerides		Glucose		HOMA-IR	
		*β*	*p*	*β*	*p*	*β*	*p*	*β*	*p*	*β*	*p*	*β*	*p*	*β*	*p*	*β*	*p*
Betaine	1	− 0.020	0.273	0.001	0.297	− 0.900	0.093	− 0.030	0.853	− 0.010	0.216	0.180	0.798	0.030	0.908	0.030	0.246
	2	− 0.016	0.376	< 0.001	0.547	− 0.790	0.170	− 0.033	0.832	− 0.010	0.392	0.350	0.649	0.069	0.802	0.026	0.351^#^
Choline	1	0.020	0.763	− 0.003	0.386	− 0.560	0.716	0.340	0.516	− 0.050	0.130	− 2.990	0.134	− 0.400	0.574	0.090	0.210
	2	0.018	0.742	− 0.004	0.319	− 1.090	0.529	0.340	0.571	− 0.052	0.111^#^	− 3.08	0.164^#^	0.520	0.492^#^	0.160	0.056^#^
Cysteine	1	0.001	0.891	0.01	0.035^*^	− 0.190	0.313	− 0.060	0.398	0.001	0.832	0.040	0.888	0.002	0.983	0.010	0.185
	2	0.002	0.755	< 0.001	0.157	− 0.320	0.184	− 0.011	0.898	− 0.006	0.166^#^	− 0.220	0.473^#^	0.045	0.680	0.014	0.222^#^
DMG	1	− 0.410	0.009^*^	0.010	0.534	− 10.80	0.031^*^	− 1.100	0.530	− 0.060	0.566	− 0.670	0.919	0.700	0.764	0.400	0.098
	2	− 0.400	0.038^*^	0.009	0.510	− 8.160	0.180	− 2.410	0.257	− 0.019	0.974^#^	− 2.46	0.760^#^	4.02	0.175	0.72	0.018^*^
Glycine	1	< 0.001	0.919	< 0.001	0.782	0.090	0.357	− 0.020	0.529	0.003	0.066	0.160	0.182	0.010	0.751	0.007	0.100
	2	− 0.007	0.841	< 0.001	0.588	0.094	0.368	− 0.014	0.677	0.003	0.129^#^	0.170	0.200	0.021	0.657	0.006	0.222
Homocysteine	1	0.010	0.922	0.020	0.008^*^	− 1.220	0.774	− 1.620	0.264	0.050	0.505	1.610	0.770	0.800	0.682	0.180	0.373
	2	− 0.002	0.990	0.020	0.042^*^	− 2.740	0.587	− 1.030	0.547	− 0.019	0.838	− 2.63	0.675^#^	1.65	0.473	− 0.350	0.140
Methionine	1	0.020	0.602	0.002	0.343	− 0.150	0.871	− 0.250	0.436	0.020	0.387	0.600	0.617	0.650	0.126	0.160	< 0.001^*^
	2	0.022	0.505	0.002	0.346	− 0.820	0.412	− 0.230	0.510	0.006	0.784	0.550	0.674	0.730	0.120^#^	0.140	0.002^*, #^
Serine	1	− 0.010	0.266	< 0.001	0.668	− 0.001	0.774	− 0.060	0.399	0.003	0.436	0.120	0.657	0.040	0.696	0.020	0.063
	2	− 0.006	0.462	< 0.001	0.576	0.062	0.848	− 0.038	0.628	0.003	0.473^#^	0.100	0.757	0.092	0.416	0.019	0.103^#^
Betaine/choline	1	− 0.320	0.201	0.030	0.104	− 10.20	0.201	− 2.830	0.305	0.030	0.837	13.80	0.186	2.430	0.512	0.190	0.614
	2	− 0.280	0.337	0.022	0.240	− 7.290	0.433	− 3.05	0.338	0.130	0.456^#^	20.6	0.084	0.062	0.988	− 0.096	0.826
DMG/betaine	1	− 7.760	0.099	− 0.120	0.700	20.70	0.891	− 58.00	0.265	6.17	0.034^*^	101	0.608	− 10.90	0.876	0.090	0.990
	2	− 9.470	0.112	0.012	0.975^#^	161	0.395	− 77.5	0.23 ^b^	8.74	0.014^*^	50.7	0.835	94.4	0.284	7.95	0.389

β estimates and *p* values presented for each metabolite fitted as an independent variable in unadjusted (Model 1) or models adjusted for age, sex, baseline metabolite status, GFR, and BMI for non-anthropometric dependent variables (Model 2). All independent and dependent variables refer to the change of each metabolite and metabolic marker (baseline—six-month follow-up). Further details for intervention effects are presented in Supplementary Table 2

Abbreviations: *DMG* dimethylglycine, *HDL-C* HDL-cholesterol, *LDL-C* LDL-cholesterol

*Indicates an association between shifts in the dependent (change in metabolite concentration) and independent (change in cardiometabolic parameter) variables

^#^Indicates a significant interaction between the metabolite and intervention group

**Fig. 2:**
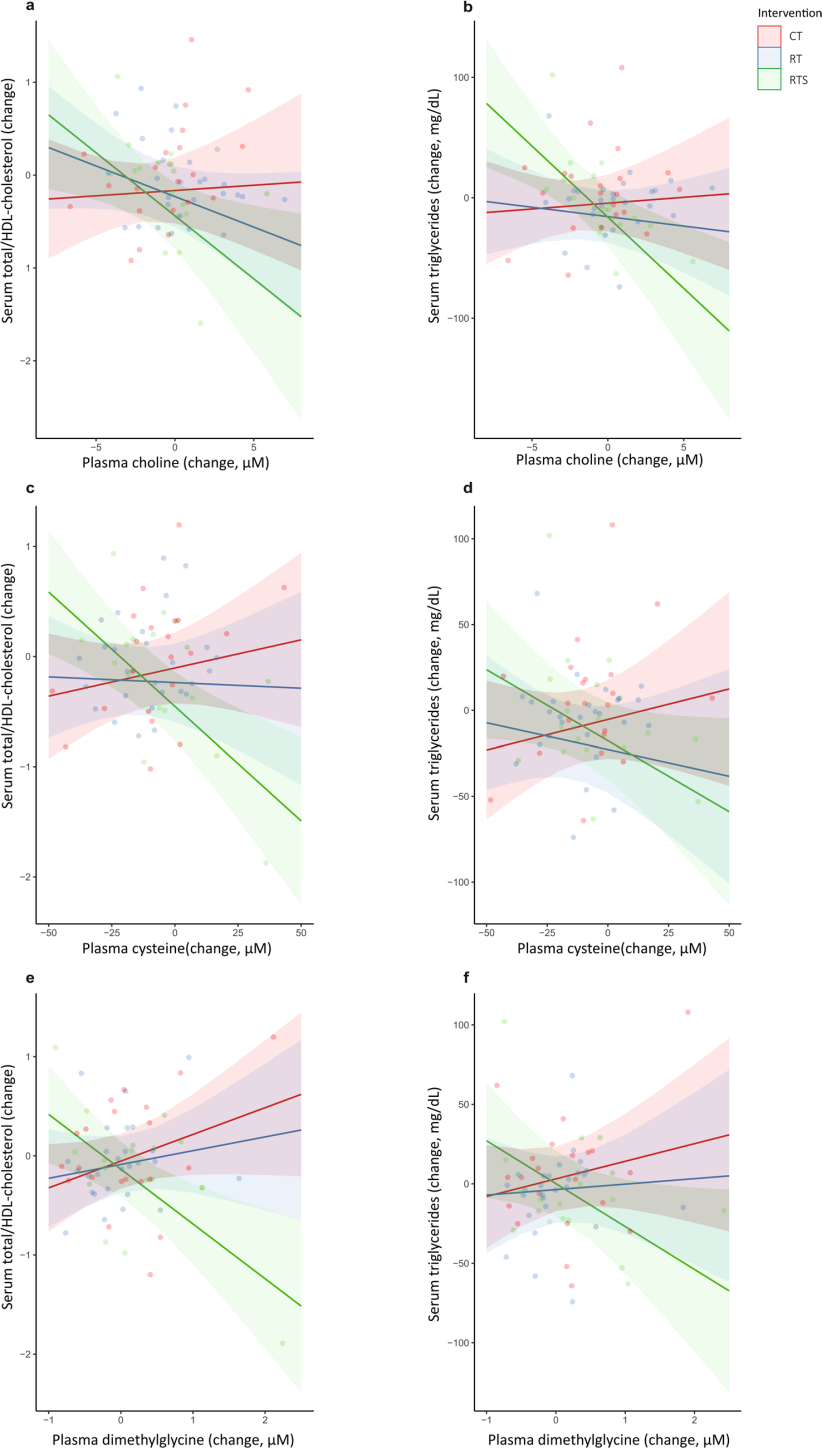
Effect of intervention with resistance training (RT), resistance training with supplementation (RTS), or cognitive training (CT) on associations between six-month shifts in one-carbon metabolites and cardiometabolic parameters. Scatter plots depict associations between changes in choline concentrations with **a** total/HDL-cholesterol and **b** triglyceride concentrations; cysteine concentrations with **c** total/HDL-cholesterol and **d** triglyceride concentrations, and dimethylglycine concentrations with **e** total/HDL-cholesterol and **f** triglyceride concentrations. Data are based on linear regression analyses presented in Table 4 and Supplementary Table 2, and refer to a model adjusted for confounding variables (age, sex, glomerular filtration rate, baseline metabolite concentrations, and BMI), and includes an interaction term between the intervention group and the dependent variable. Change (baseline—follow-up) in metabolites are set as dependent variables, and change in cardiometabolic markers are set as independent variables

The most consistent effect of the intervention was seen in markers of lipid metabolism. In the RTS group, increasing concentrations of choline, cysteine, DMG, glycine, and serine were associated with a decline in the ratio of total cholesterol/HDL-C compared to the CT and RT groups. Similarly, increasing concentrations of choline, cysteine, DMG, and methionine were associated with reduced triglyceride concentrations (interaction, *p* < 0.05) in the RTS group compared to the CT and RT groups. Rising betaine and serine concentrations were also associated with a decline in HOMA-IR in the RTS group compared to the CT and RT groups, while positive associations between shifts in methionine and cysteine with HOMA-IR were found in both the RTS and RT groups compared to those in the CT group (Fig. 2). Full details of these effects can be found in Supplementary Table 2.

## Discussion

This study is a novel investigation into the impact of lifestyle interventions on a comprehensive profile of 1C metabolites and cardiometabolic parameters in an elderly population (65–98 years). Metabolites reflecting upregulated betaine-dependent Hcy remethylation were associated with an unfavourable lipid profile at baseline. However, increasing DMG, cysteine, and choline concentrations were associated with improved lipid parameters in those receiving nutritional supplementation, which likely reflects underlying shifts in methylation status and choline availability, both of which play a critical role in lipid metabolism. This study highlights the complex relationship between 1C metabolism and cardiometabolic health, which choline metabolites appear central to. Notably, our findings highlight that the relationship between 1C metabolites and cardiometabolic health may be modified by resistance training combined with nutritional supplementation. These findings bolster the relevance of 1C metabolites in healthy ageing, and further research is required to understand whether optimising 1C metabolite status might confer cardiometabolic and related benefits in very old adults.

Hcy is an established risk factor for cardiovascular disease [7]. Despite growing interest in the association between Hcy and the metabolic syndrome, this has yielded inconsistent findings and is poorly characterised [8, 42–46]. While associations between Hcy and cardiometabolic markers were limited, metabolites reflective of betaine-dependent Hcy remethylation were more closely associated with parameters of glucose and lipid metabolism. Our findings indicate that upregulation of the BHMT pathway to support Hcy remethylation is associated with an unfavourable cardiometabolic profile; betaine and the ratio of betaine/choline were inversely correlated with HOMA-IR and triglycerides, while DMG and the ratio of DMG/betaine were positively correlated with triglycerides, total/HDL-C and HOMA-IR. Shifts in DMG concentrations were associated with changes in cardiometabolic markers, yet the direction of response was inconsistent, as increasing DMG concentrations were associated with improvements in LDL-C and BMI, but worsening insulin sensitivity in adjusted models. These findings in part support previous reports of betaine being associated with a more favourable cardiometabolic profile, although based on the available evidence, we would have expected to find a more divergent association between betaine and choline with these metabolic markers rather than between betaine and DMG [12–15]. There is a relative paucity of literature regarding DMG, although lower concentrations have previously been associated with higher blood glucose, increased insulin resistance, and an increased risk of incident diabetes [47]. The only known pathway for DMG synthesis results from betaine donating a methyl group for the remethylation of Hcy, and thus is considered to reflect BHMT activity [48, 49]. Upregulation of this pathway is evidently implicated in numerous aspects of cardiometabolic risk, which, for the most part, appears to be an unfavourable relationship.

Interestingly, supplementation modified the longitudinal relationship between shifts in these markers of Hcy remethylation and lipid metabolism. Increasing DMG, choline, and cysteine concentrations were associated with improvements in the lipid profile only in those receiving supplementation. Although we are unable to provide further insight into mechanisms, this is plausibly mediated by shifts in methylation status and choline availability for the synthesis of phosphatidylcholine. With increased flux through BHMT, phosphatidylcholine synthesis from choline is restricted, as there is greater demand for choline oxidation to support betaine availability. In turn, this places an increased demand on the synthesis of phosphatidylcholine from phosphatidylethanolamine, which requires labile methyl groups [50]. Indeed, betaine supplementation has been shown to increase hepatic phosphatidylcholine concentrations and the ratio of phosphatidylcholine/phosphatidylethanolamine [51]. In another study, betaine supplementation improved whole-body glucose homeostasis and energy expenditure [52]. Supplementation with krill oil has been shown to reduce triglycerides, which was suggested to be through increased phosphatidylcholine intake (1750 mg/day) and choline availability leading to enhanced processes dependent on methyl donors, including flux over BHMT [53]. The Fortifit supplement used in the current study contains only a small amount of choline (55 mg). Although B vitamin supplementation may increase choline status [54], greater supplemental concentrations, or a longer follow-up period may have been required to see similar improvements in the lipid profile of our participants.

While increasing cysteine concentrations were associated with favourable changes in lipid parameters in those receiving supplementation, this should be considered alongside the relationship between cysteine and body composition. Cysteine concentrations were correlated with higher BMI and waist/hip ratio at baseline. Increasing cysteine concentrations were also associated with a rise in waist/hip ratio during the six-month follow-up in simple regression analyses regardless of the intervention received, but not when adjusted for confounding variables. These findings in part align with those from the Hordaland Homocysteine study [55, 56]*,* where cysteine was positively associated with BMI and fat mass in both cross-sectional and longitudinal analyses in a large cohort of middle-aged and older adults [10]. Beyond cysteine’s link to methylation status, mechanisms have been extensively reviewed and proposed by Elshorbagy et al. [10]. Our findings support the role of cysteine in body composition, but require careful interpretation in elderly populations, as there is a shift upwards in the BMI range that is considered healthy and protective against mortality in older adults [57]. Vitamin B_6_ is required for cysteine synthesis (through cystathionine-*β*-synthase and cystathionine-*λ*-lyase), yet vitamin B_6_ deficiency is prevalent amongst elderly populations [58, 59]. The balance is complex, and further studies are required to better understand the mechanisms underlying the role of cysteine in the regulation of body composition and lipid metabolism, particularly in vulnerable populations that require special consideration, such as those in their oldest years.

It is interesting to note that Hcy did not decline in those receiving the RT or RTS intervention, an observation previously been reported with both B vitamin supplementation [60, 61] and RT [19, 20] in older adults. This might be explained by the nutritional supplement used here, which contained a moderate amount of 200 µg folic acid per serve compared to 400 µg shown to reduce Hcy in other elderly populations [60, 61]. The Fortifit supplement also provided 20.7 g of protein per serve, thus increasing the supply of dietary methionine. Further, participants in the current study were older (mean, 83 ± 6 years) than other elderly populations who have shown a decline in Hcy with RT interventions (range 60–80 years) [19, 20]. However, caution should be given to the interpretation of these findings given the analytical technique used. While the mass spectrometry technique used has been peer-reviewed with good internal reproducibility [36], the concentrations of Hcy are below expected reference ranges [63] which limits the clinical interpretation of our results. The discrepancy in Hcy concentrations can be explained by a lower concentration of Tris (2-carboxyethyl) phosphine used, an agent used in sample preparation to reduce disulfide bonds in cystine and homocystine to allow quantification of cysteine and Hcy, compared to other studies [64].

This study provides valuable insight into the response of 1C metabolism to intervention those in their oldest years of life. A key strength of our study lies in the elderly population included (83 ± 6 years). Octogenarians and nonagenarians are the fastest growing proportion of our ageing population [65], yet are under-represented in research on 1C metabolism. Lifestyle interventions, such as supplementation and exercise, are promoted to optimise health in this vulnerable population, yet to our knowledge, this is the first study to investigate the effect of such interventions on cardiometabolic parameters in advanced age. These results, of course, should not be generalised to early agers where B vitamin status might be expected to be more adequate than in our cohort. Further, while the mass spectrometry technique used here gave a comprehensive profile of plasma 1C metabolites, but does not quantify sarcosine, trimethylamine *N*-oxide, or other choline species (e.g. phosphatidylcholine). The inclusion of these metabolites would help to interpret the findings in the current study, as would quantifying urinary 1C metabolites [66].

## Conclusion

This study highlights the complex relationship between 1C metabolites and cardiometabolic health in an elderly institutionalised population, which choline metabolites were central to. While RT or RTS did not improve plasma 1C metabolites or cardiometabolic risk profiles after six months, more subtle changes in pathway regulation were indicated. 1C metabolites reflecting upregulated betaine-dependent Hcy remethylation were correlated with an unfavourable cardiometabolic profile. However, the direction of association between these 1C metabolites and the lipid profile was modified in those receiving RT with nutritional supplementation. These findings highlight the potential to promote successful ageing through improving health outcomes underpinned by these interconnected pathways, however, further research is required to demonstrate the potential of optimising 1C metabolite status such that cardiometabolic benefit is conferred in older adults.

## Supplementary Information

Below is the link to the electronic supplementary material.

Supplementary file1 (PDF 184 KB)
