# Supplementary material for: Nutritional supplementation alters associations between one-carbon metabolites and cardiometabolic risk profiles in older adults: a secondary analysis of the Vienna Active Ageing Study
Source: Eur J Nutr. 2021 Jul 8;61(1):169–82. doi: 10.1007/s00394-021-02607-y (PMC8783863; doi:10.1007/s00394-021-02607-y)
Supplement: Supplementary file 1 — Supplementary file1 (PDF 184 KB) [file 394_2021_2607_MOESM1_ESM.pdf]

Supplementary Material: European Journal of Nutrition

Nutritional supplementation alters associations between one-carbon metabolites and cardiometabolic risk profiles in older adults: A secondary analysis of the Vienna Active Ageing Study.

Nicola A. Gillies, Bernhard Franzke, Barbara Wessner, Barbara Schober-Halper, Marlene Hofmann, Stefan Oesen, Anela Tosevska, Eva-Maria Strasser, Nicole C. Roy; Amber M. Milan, David Cameron-Smith, Karl-Heinz Wagner*

* Corresponding author:
Professor Karl-Heinz Wagner
Platform Active Ageing, Univeristy of Vienna
Althanstrasse 14, 1090 Vienna, Austria
Email: karl-heinz.wagner@univie.ac.at

Supplementary Table 1: Nutritional composition of the Fortifit supplement 2

Supplementary Table 2: Effect of intervention with resistance training with or without nutritional supplementation compared to control on the association between changes in one-carbon metabolites and cardiometabolic parameters from baseline to six-month follow-up..................................3

Supplementary Tale 1 – Nutritional composition of the Fortifit supplement

Nutrient		Per 150 mL serve
Energy		150 kcal
Protein	Total	20.7g
	Whey Protein	19.7g
	Essential amino acids (including leucine)	>10g
Carbohydrates	Total	9.4g
	Sugars	4.2g
	Dietary Fibre	
Fat	Total	3.0g
	Saturated fat	0.8g
Vitamins	Vitamin A	150 µg RE
	Vitamin C	32 mg
	Vitamin D3	20 µg
	Vitamin B1	225 µg
	Vitamin B2	250 µg
	Vitamin B3	2.7 mg NE
	Pantothenic Acid	800 µg
	Vitamin B6	750 µg
	Folic acid	200 µg
	Vitamin B12	3.0 µg
	Biotin	6.0 µg
	Vitamin E	7.5 mg α-TE
	Vitamin K	11 µg
Minerals	Calcium	500 mg
	Chloride	70 mg
	Chromium	7.4 µg
	Copper	270 µg
	Fluoride	150 µg
	Iodine	20 µg
	Iron	2.4 mg
	Magnesium	37 mg
	Manganese	500 µg
	Molybdenum	14 µg
	Phosphorus	250 mg
	Potassium	270 mg
	Selenium	15 µg
	Sodium	150 mg
	Zinc	2.2 mg
Others	Carotenoids	0.3 mg
	Choline	55 mg

The Fortifit supplement (Nutricia) was consumed 9 times a week during the intervention (daily, with two occasions where the supplement was consumed following resistance training sessions). Abbreviations: NE, niacin equivalents; RE, retinol equivalents; TE, tocopherol equivalents.

Supplementary Table 1 – Effect of intervention with resistance training with or without nutritional supplementation compared to control on the association between changes in one-carbon metabolites and cardiometabolic parameters from baseline to six-month follow-up

		BMI		Waist/Hip ratio		LDL-C		HDL-C		Total/HDL-C		Triglycerides		Glucose		HOMA-IR	
Metabolite		β	*p*	β	*p*	β	*p*	β	*p*	β	*p*	β	*p*	β	*p*	β	*p*
Betaine	RT	-0.06	0.183	<0.01	0.157	-0.56	0.706	-0.75	0.136	0.03	0.360	0.77	0.691	-0.94	0.175	-0.07	0.326
	RTS	-0.02	0.650	<0.01	0.385	-1.29	0.370	0.20	0.684	-0.03	0.220	-0.88	0.639	-0.82	0.216	-0.17	0.013[#]
Choline	RT	-0.11	0.239	<0.01	0.971	-3.85	0.205	-0.06	0.956	-0.07	0.178	-2.51	0.485	-3.14	0.015[#]	-0.34	0.016[#]
	RTS	<0.01	0.944	-0.01	0.320	-2.24	0.530	0.85	0.484	-0.15	0.025[#]	-12.8	0.004[#]	-2.21	0.135[†]	-0.21	0.180[†]
Cysteine	RT	<0.01	0.467	<0.01	0.233	<0.01	0.976	0.11	0.488	<0.01	0.490	-0.65	0.259	-0.15	0.481	-0.05	0.013[#]
	RTS	<0.01	0.314	<0.01	0.705	-0.52	0.249	0.27	0.083	-0.03	0.002[#]	-1.16	0.039[#,§]	-0.29	0.162	-0.06	0.005[#]
DMG	RT	-0.05	0.910	-0.02	0.541	11.4	0.398	3.97	0.395	-0.13	0.594	-7.58	0.662	-6.50	0.299	-0.66	0.302
	RTS	-0.19	0.627	0.04	0.183	-10.2	0.419	7.70	0.081	-0.81	<0.001[#,‡]	-37.8	0.023[#]	0.56	0.924	-0.90	0.137
Glycine	RT	<0.01	0.621	<0.01	0.510	-0.21	0.355	<0.01	0.194	-0.01	0.016[#]	-0.55	0.052	-0.10	0.310	-0.02	0.067
	RTS	<0.01	0.174	<0.01	0.993[†]	-0.48	0.060	0.16	0.067	-0.02	<0.001[#]	-0.61	0.055	-0.14	0.227	-0.02	0.152
Homocysteine	RT	0.410	0.309	0.02	0.405	11.3	0.376	1.12	0.794	0.06	0.810	0.47	0.976	2.94	0.598	-0.57	0.331
	RTS	-0.02	0.959	<0.01	0.930	9.32	0.483	1.67	0.710	-0.09	0.709	-6.21	0.705[§]	-3.69	0.522	-0.87	0.150
Methionine	RT	-0.12	0.101	0.01	0.191	-2.03	0.386	-0.40	0.625	-0.04	0.377	-3.65	0.223	-2.29	0.030[#]	-0.34	0.001[#]
	RTS	-0.12	0.134	-0.01	0.214	-1.39	0.579	0.44	0.610	-0.08	0.084	-6.57	0.044[#]	-0.29	0.011[#]	-0.28	0.007[#]
Serine	RT	-0.02	0.289	<0.01	0.271	0.12	0.812	<0.01	0.560	-0.01	0.347	-0.94	0.152	-0.20	0.382	-0.04	0.071
	RTS	<0.01	0.507	<0.01	0.488	-0.51	0.425	0.21	0.318	-0.03	0.021[#]	-0.73	0.364	-0.16	0.567	-0.06	0.029[#]
Betaine/choline	RT	0.32	0.712	0.07	0.171	37.3	0.164	-8.27	0.367	1.25	0.012[#]	34.7	0.306	10.3	0.382	0.95	0.431
	RTS	0.27	0.753	0.07	0.160	12.0	0.649	3.74	0.678	0.65	0.175	50.4	0.135	-0.16	0.567	-1.31	0.273[†]
DMG/betaine	RT	-1.77	0.919	-3.13	0.002[#]	710	0.203	400	0.030[#]	-9.29	0.354	-860	0.217	10.3	0.382	1.77	0.947
	RTS	-18.7	0.295	0.01	0.928	270	0.639	170	0.363	-9.60	0.352[‡]	-1200	0.098	-2.56	0.826[†]	17.4	0.531

β estimates and *p* values are presented for the interaction term fitted between the independent variable (change in metabolite concentration from baseline to six-month follow-up) and intervention group (receiving the resistance training or resistance training with supplementation intervention compared to the control group). Data presented from each model is adjusted for age, sex, baseline metabolite status, GFR, and BMI (non-anthropometric variables). [#] Indicates a significant difference according to intervention, [†] Indicates that the dependent variable is inversely associated with baseline metabolite status in the corresponding model, [‡] Indicates that the dependent variable is lower in males in the corresponding model, [§] Indicates that the dependent variable is positively associated with baseline metabolite status in the corresponding model. Abbreviations: DMG, dimethylglycine; HDL-C, HDL-cholesterol; LDL-C, LDL-cholesterol.
